# Systematic evaluation of antibody-mediated siRNA delivery using an industrial platform of THIOMAB–siRNA conjugates

**DOI:** 10.1093/nar/gku1362

**Published:** 2014-12-30

**Authors:** Trinna L. Cuellar, Dwight Barnes, Christopher Nelson, Joshua Tanguay, Shang-Fan Yu, Xiaohui Wen, Suzie J. Scales, Julie Gesch, David Davis, Anja van Brabant Smith, Devin Leake, Richard Vandlen, Christian W. Siebel

**Affiliations:** 1 Department of Molecular Biology, Genentech, Inc., 1 DNA Way, South San Francisco, CA 94080-0511, USA; 2 Department of Discovery Oncology, Genentech, Inc., 1 DNA Way, South San Francisco, CA 94080-0511, USA; 3 Department of Protein Chemistry, Genentech, Inc., 1 DNA Way, South San Francisco, CA 94080-0511, USA; 4 Dharmacon Products, Thermo Fisher Scientific, 2650 Crescent Drive, Suite 100, Lafayette, CO 80026, USA

## Abstract

Delivery of siRNA is a key hurdle to realizing the therapeutic promise of RNAi. By targeting internalizing cell surface antigens, antibody–siRNA complexes provide a possible solution. However, initial reports of antibody–siRNA complexes relied on non-specific charged interactions and have not been broadly applicable. To assess and improve this delivery method, we built on an industrial platform of therapeutic antibodies called THIOMABs, engineered to enable precise covalent coupling of siRNAs. We report that such coupling generates monomeric antibody–siRNA conjugates (ARCs) that retain antibody and siRNA activities. To broadly assess this technology, we generated a battery of THIOMABs against seven targets that use multiple internalization routes, enabling systematic manipulation of multiple parameters that impact delivery. We identify ARCs that induce targeted silencing *in vitro* and extend tests to target prostate carcinoma cells following systemic administration in mouse models. However, optimal silencing was restricted to specific conditions and only observed using a subset of ARCs. Trafficking studies point to ARC entrapment in endocytic compartments as a limiting factor, independent of the route of antigen internalization. Our broad characterization of multiple parameters using therapeutic-grade conjugate technology provides a thorough assessment of this delivery technology, highlighting both examples of success as well as remaining challenges.

## INTRODUCTION

A groundbreaking discovery in 1998, RNA interference (RNAi) describes the fundamental process in eukaryotes in which double-stranded (ds) RNAs induce the cleavage of mRNAs with complementary sequences (1). RNAi has been embraced as an innovative therapeutic modality that holds promise to revolutionize therapy for numerous human diseases. Small interfering RNA (siRNA) drugs can be designed and produced far more efficiently and quickly than can small molecule or protein drugs. Moreover, siRNAs pave the way to ‘drugging the un-druggable’, that is, to generating therapeutic inhibitors of proteins (e.g. transcription factors) that are recalcitrant to current drug development technologies. The main hurdle to realizing the therapeutic promise of RNAi is the safe and effective systemic delivery of siRNA.

Early clinical trials of siRNA-based drugs employed local delivery, such as direct injection into the vitreous humor of the eye or systemic administration of lipid-based vehicles that primarily deliver to the liver (2). Subsequent clinical trials have employed systemic methods to deliver siRNAs against cancer targets, and these trials stand as major milestones in the field. The first such trial relied on nanoparticle carriers and reported silencing in non-liver tumors, and a more recent trial, using lipid nanoparticles, reported regression of liver metastases in endometrial cancer patients (3,4). A remaining important challenge for the next generation of delivery vehicles is to develop a safe method that supports not only systemic administration but also targets siRNAs to specific tumor cell types, moving beyond the non-specific accumulation of non-targeted nanoparticles in tumors that appears to be due to the enhanced permeability and retention effect (EPR) (5,6). As an important advance on this goal, transferrin has been used as a targeting agent of siRNA-loaded nanoparticles. Transferrin targeting indeed increases siRNA delivery, although these nanoparticles also accumulate in non-targeted tissues, specifically the liver and kidneys (6). Toward further understanding targeted, systemic delivery methods, we set out to assess the targeting efficiency and specificity of a platform of antibody-directed conjugates.

Given their ability to bind antigens with a high degree of specificity and their established use as therapeutics, monoclonal antibodies have been tested as targeting agents (7), a rationale bolstered by the clinical successes of antibody–drug conjugates (8). Indeed, a number of groups have described RNAi silencing using antibody–siRNA complexes to deliver siRNA into the cell via internalization of targeted cell surface antigens (9–11). However, generating these complexes relied not on direct antibody-siRNA conjugation but rather on nonspecific electrostatic interactions between highly positively charged peptides and the negatively charged siRNA, resulting in heterogeneous aggregates. Heterogeneity of drug loading onto antibodies affects clearance, maximum tolerated dose, and efficacy (12). While these initial reports represent an intriguing proof-of-concept test of antibody-mediated siRNA delivery, a multitude of significant improvements are required to generate antibody-based vehicles that meet the rigorous demands of drug manufacturing and clinical trials. Since the heralding of these first reports 8 years ago, publications of improvements have been sparse and such antibody-siRNA complexes have yet to advance into the clinic.

We aimed to improve antibody–siRNA delivery by (a) developing homogenous, pure antibody–siRNA conjugates using technologies amenable to drug manufacturing and (b) testing how multiple parameters affect siRNA delivery and silencing. We built on our THIOMAB technology (13,14), established for use in the clinic with antibody–drug conjugates, to covalently attach chemically stabilized siRNAs to discrete positions on the antibody backbone with a defined antibody:siRNA stoichiometry. We further describe the systematic study of the antibody siRNA delivery platform, using antibodies against seven different cell surface receptors that utilize various routes of internalization, while also varying linker chemistry, linkage position and antibody format. We demonstrate that both the siRNA and antibody components of the ARCs maintain normal function in the conjugate and, most importantly, that some ARCs can deliver siRNAs into cells to induce competent silencing that is dependent on covalent coupling and antigen expression, including *in vivo* using preclinical tumor models. Our characterization also reveals that important challenges remain to fully enable this delivery platform, particularly improving release of siRNA from endosomal compartments.

## MATERIALS AND METHODS

### siRNAs and antibody–siRNA conjugates

For the description of ARC generation refer to Tan *et al.* (15). The siSTABLE PPIB sequence was stabilized using 2′-*O*-methylated bases (denoted by m) and 2′-fluoro bases (denoted by 2′-F) modifications. The sequences are: sense: 5′-DY547.mA.mC.A.G.mC.A.A.A.mU.mU.mC.mC.A.mU.mC.G.mU.G.mU.N6-3′, antisense: 5′-P.A.2′-F-C.A.2′-F-C.G.A.2′-F-U.G.G.A.A.2′-F-U.2′-F-U.2′-F-U.G.2′-F-C.2′-F-U.G.2′-F-U.U.U-3. The chemically stabilized siSTABLE NTC sequences are: sense: 5′-DY547.mG.mA.mU.mU.A.mU.G.mU.mC.mC.G.G.mU.mU.A.mU.G.mU.A.N6-3′, antisense: 5′-P.2′-F-U.A.2′-F-C.A.2′-F-U.A.A.2′-F-C.2′-F-C.G.G.A.2′-F-C.A.2′-F-U.A.A.2′-F-U.2′F-C.U.U-3′. The chemically stabilized siSTABLE, PPIB-mismatch (PPIBmm) control sequence is: sense: 5′-DY547.mA.mC.A.G.mC.A.A.A.A.A.G.mC.A.mU.mC.G.mU.G.mU.N6-3′, antisense: 5′-P.A.2′-F-C.A.2′-F-C.G.A.2′-F-U.G. 2′-F-C.2′-F-U.2′-F-U.2′-F-U.2′-F-U.2′-F-U.G.2′F-C.2′F-U.G.2′F-U.U.U -3. siRNAs were generated with and without Dy547 (position is noted above) for tracking studies. For HPS4 work, nine different siRNA reagents were obtained from Dharmacon: siGenome HPS4 Pool and HPS4 siRNAs (all four available), as well as the ON-Target Plus (OTP) HPS4 siRNAs (set of 4). The siGenome pool, siGenome HPS4-3, and OTP HPS4-8 gave the strongest phenotypic effects, knocking down both RNA and HPS4 protein.

### ARC mass measurements and calculations

Immunoglobulin monomers have a molecular mass of ∼150 kD (e.g. Trastuzumab = 149 kD, anti-TENB2 = 148 kD and anti-NaPi2b = 151 kD). The 21-mer siRNAs we employed have a molecular mass of ∼1/10th that of an immunoglobulin monomer (e.g. siPPIB3 without dye labeling = 13 kD). Thus, ARCs carrying one or two siRNAs have a molecular mass of ∼163 and 176 kD, respectively. Protein concentrations of the purified ARCs were determined by the BCA assay, and siRNA stoichiometry was determined by mass spectrometry.

### Quantigene and Taqman assays

We followed the manufacturer's protocol for Panomics QuantiGene 2.0 assays, normalizing target mRNA levels to the housekeeping genes GAPDH, β-actin or RPLP0. For Taqman assays, RNA was harvested using TRIzol (Life Technologies) or RNeasy (Qiagen). Total RNA was reverse transcribed and amplified by qPCR with Taqman FAM MGB probes. The data were analyzed using the ddCt (2^−ΔΔCT^) method and normalized to glyceraldehyde 3-phosphate dehydrogenase (GAPDH) mRNA unless noted otherwise (16). To generate bar graphs for gene silencing experiments, biological replicates were first normalized to the appropriate NTC ARC, and then the data were combined. Error bars represent the standard deviation between experiments.

### *In vitro* silencing assays

Cells were seeded into 96- or 24-well plates the day before experiments. ARCs were added to cells at noted concentrations in complete media the following day and typically allowed to incubate on cells for 72 h, before silencing analysis was performed (unless noted otherwise). For positive control transfections, we used Dharmafect 2 transfection reagents, which efficiently transfects PC3, 293 and Igrov-1 cell lines. ARCs were transfected as positive controls with standard methods used for siRNAs.

For the mini-screen, cells were first treated with siRNAs to knockdown endocytic pathway genes for 24 or 72 h. At this point, cells were washed, ARCs were added for an additional 72 h, and silencing analyses were performed.

### *In vitro* imaging assays

Cell lines stably transfected with the indicated antigens were pre-plated onto on eight-well slides and incubated with 5 μg/ml ARCs for 30 min on ice, then washed and chased for 40 h at 37°C in the presence (continuous uptake) or absence (pulse-chase) of lysosomal protease inhibitors. Continuous uptake experiments were performed by adding ARCs onto the cells for noted times without washing. Cells were fixed in 3% paraformaldehyde (PFA, Sigma) for 20 min, permeabilized with 0.4% saponin (Sigma) in phosphate-buffered saline (PBS) with 1% bovine serum albumin (BSA) (Sigma), and antibodies were detected with FITC anti-human antibody (Jackson) while siRNAs were imaged with Dy547 or Dy647. Lysosomes were labeled with mouse anti-LAMP1 (BD555798, 1:1000), and Cy5-anti-mouse (Jackson), and P-bodies were labeled with anti-Dcp1a (Novus H00055802-A01, 1:600) followed by FITC-anti-mouse (Jackson).

### Flow cytometry

Cell lines stably transfected with the indicated antigens were detached with ethylenediaminetetraacetic acid (EDTA)-PBS, washed, and incubated with unlabeled primary antibodies or ARCs at 5 μg/ml for 30 min on ice. Cells were washed twice with FACS buffer (PBS, pH 7.2, 0.5% BSA, 2 mM EDTA) and incubated with Alexa488 goat anti-human antibody (1:2000, H+L, Life Technologies A-11013). Cells were then washed three times, stained with propidium iodide (500 ng/mI) and analyzed on a FACSCalibur (BD Biosciences).

### 5′ RACE

Total RNA was isolated with TRIzol (Life Technologies). Invitrogen gene racer kits were used to perform 5′ RACE assays to detect RNAi cleavage fragments. The de-capping step was omitted to facilitate capture of AGO2 cleaved mRNAs with a free 5′ phosphate. The following oligonucleotides were used for PCR and nested PCR: GeneRacer 5′ primer and reverse gene-specific primer for PPIB: 5′ CTCTCCACCTTCCGCACCACCTCCA. For nested PCRs, the GeneRacer 5′ nested primer and reverse nested gene-specific primer for PPIB: 5′ TCTTTGCCTGCGTTGGCCATGCTCAC.

### Western blotting

Protein lysates were generated with radioimmunoprecipitation assay buffer (RIPA, Thermo Scientific 89900) including Roche mini-complete protease inhibitor tablets, and protein concentrations were determined using BCA assays (Pierce). Samples were prepared using NuPAGE reagents (Invitrogen) and electrophoresed using NuPAGE Novex 4–12% Bis–Tris protein gels (Life Technologies). Samples were transferred onto Hybond ECL low-fluorescent nitrocellulose paper (Amersham) via a traditional wet-transfer for 1 h. Membranes were blocked overnight with 2% ECL advance blocking agent (Amersham) in Tris-buffered saline with 0.05% Tween 20 (TBS-T). The following antibodies were used: Abcam ab16045 rabbit anti-PPIB (1:1000), Novus 2D4A6 mouse anti-GAPDH (1:20 000), ECL Plex Cy5 anti-rabbit IgG (1:1500) and ECL Plex Cy3 anti-mouse IgG (1:1500). Membranes were imaged using a Typhoon TRIO imager (Amersham), with intensities quantified using ImageJ spot densitometry.

### *In vivo* study design and isolation of CFSE/EpCAM-positive tumor cells

*nu/nu* (Charles River) mice were inoculated in the right flank with 5 million PC3-TENB2-high cells in a volume of 0.1 ml Hank's Balanced Salt Solution (HBSS) per mouse. Mice were randomly grouped when the mean tumor size reached 200 mm^3^ (delivery study) or 400 mm^3^ (silencing study). Animals in each group were injected intravenously with 24 mg of ARC (total mass, antibody plus siRNA) per kilogram body weight on days 0, 2 and 3. Clinical observations and weight measurements were performed throughout the study and tumors were harvested on day 5. Approximately 45 min before takedown, mice were injected intravenously with 0.1 mg carboxyfluorescein diacetate succinimidyl ester (CFSE) in 50 μl dimethyl sulfoxide (DMSO), to label tumor cells surrounding blood vessels. Tumors were dissociated according to the MACS (Miltenyi Biotec, catalog #130-095-929) human tumor dissociation kit protocol. The single cell suspensions were treated with 5 ml ACK lysing buffer (Lonza, catalog #10-548E) for 1 min to remove red blood cells. After pelleting, the cells were washed with 5 ml of FACS buffer (PBS + 2% FBS). Cells were resuspended in 100 μl FACS buffer and labeled with 10 μl APC-anti-EpCAM (BD 347200) for 30 min in the dark, washed twice with 200 μl FACS buffer, filtered through a 70 μm cell strainer and then sorted on a FACS Aria high speed cell sorter (BD Biosciences).

### Confocal imaging of tumors

Tumors were drop-fixed in 4% paraformaldehyde overnight and cryoprotected in 30% sucrose for 2 days at 4°C, followed by freezing on dry ice in Tissue-Tek optimum cutting temperature (OCT) compound. Tumors were cryosectioned at 10 μm and placed at −80°C. Sections were washed with PBS and treated with Prolong Gold with 4',6-diamidino-2-phenylindole (DAPI) (Life Technologies). For co-detection of delivered antibody or CD31, slides were treated with a Super PAP Pen (Life Technologies 00-8899) and blocked with PBS + 5% goat serum and 0.1% Triton X-100. Slides were incubated with primary antibody overnight, followed by washing with PBS + 0.1% Triton X-100 and incubation with secondary antibodies for 1 h. Slides were washed and mounted with Prolong Gold containing DAPI. Antibodies used were: BD-Pharmingen 550274 PECAM-1 (1:100), A11013 goat Alexa 488-anti-rat (1:200) and A11006 goat Alexa 488-anti-human (1:200). Slides were imaged using a Leica SPE confocal microscope.

## RESULTS

### Synthesis and purification of well-defined antibody–siRNA conjugates (ARCs)

**Figure 1.**
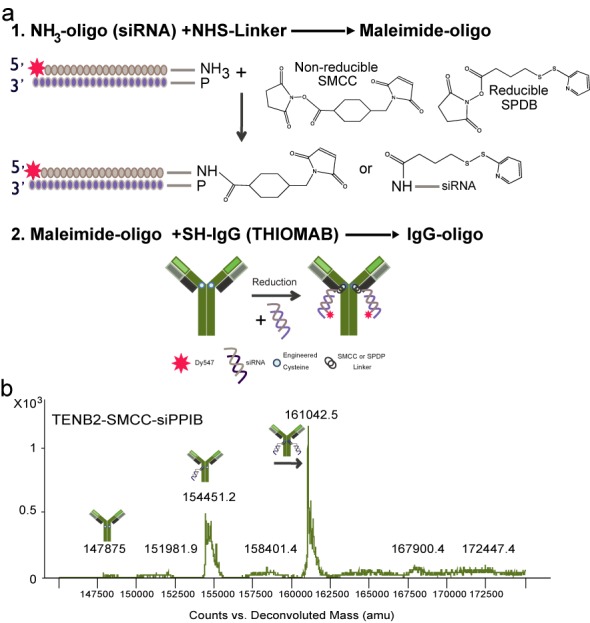
Synthesis and purification of antibody–siRNA conjugates (ARCs). (**a**) Chemically stabilized siRNAs containing a 3′ amine for coupling and a 5′ Dy547 for tracking (both modifications on the sense strand) were reacted with SMCC (non-reducible) or SPDB (reducible) NHS-linkers to form a thio-reactive adduct (maleimide-oligo). The siRNAs were then reacted with THIOMABs, containing engineered cysteines on the heavy chains (shown) or light chains to form the ARCs. (**b**) Example of electrospray TOF Mass spectrometry analysis of ARCs (after purification by anion exchange to remove free siRNA and size-exclusion chromatography to remove free antibodies). Purification yields preparations that are free of uncoupled antibodies (mass at 147875) and contain conjugates with either one siRNA (ARC mass at 154451.2) or two siRNAs (ARC mass at 161042.5). In this example, the ratio of ARCs with one siRNA to ARCs with two siRNAs is ∼1:1.

Our first goal was to determine whether pure, homogeneous preparations of an ARC could be generated and, if so, whether covalently coupling the antibody and the siRNA affected the relevant functional properties of each component. Our initial syntheses employed (i) an engineered anti-TENB2 (TMEFF2) antibody in which a cysteine residue had been introduced at a previously determined position (A118C) in the heavy chain (i.e. an anti-TENB2 HC THIOMAB), thus providing two discrete positions (one per heavy chain) for siRNA coupling, (ii) chemically stabilized siRNA (siSTABLE chemistry) modified with a 3′ amine for coupling to the passenger strand with a sequence targeting peptidlyprolyl isomerase B (PPIB, cyclophilin B) and (iii) reducible *N*-succinimidyl-4-(2-pyridyldithio)butyrate (SPDB) or non-reducible succinimidyl-4-[*N*-maleimidomethyl]cyclohexane-1-carboxylate) (SMCC) NHS (*N*-hydroxysuccinimide) linkers. ARCs were generated in two primary steps: the amine-tagged siRNA was reacted with a NHS-linker to form a thiol-reactive siRNA-linker adduct, and this adduct was then reacted with thiol groups on the THIOMAB to covalently link the siRNA via a thio-ester bond (Figure 1a). ARCs were purified using anion exchange chromatography to remove free siRNA and then by size-exclusion chromatography to remove un-coupled antibody. Gel electrophoresis (Supplementary Figure S1) and electrospray TOF mass spectrometry (Figure 1b) revealed that our synthesis and purifications yielded monomeric conjugates with one or two (average 1.67 in ARC population) covalently linked siRNAs per antibody (Figure 1b).

### Antibody and siRNA components function normally when coupled in ARCs

**Figure 2.**
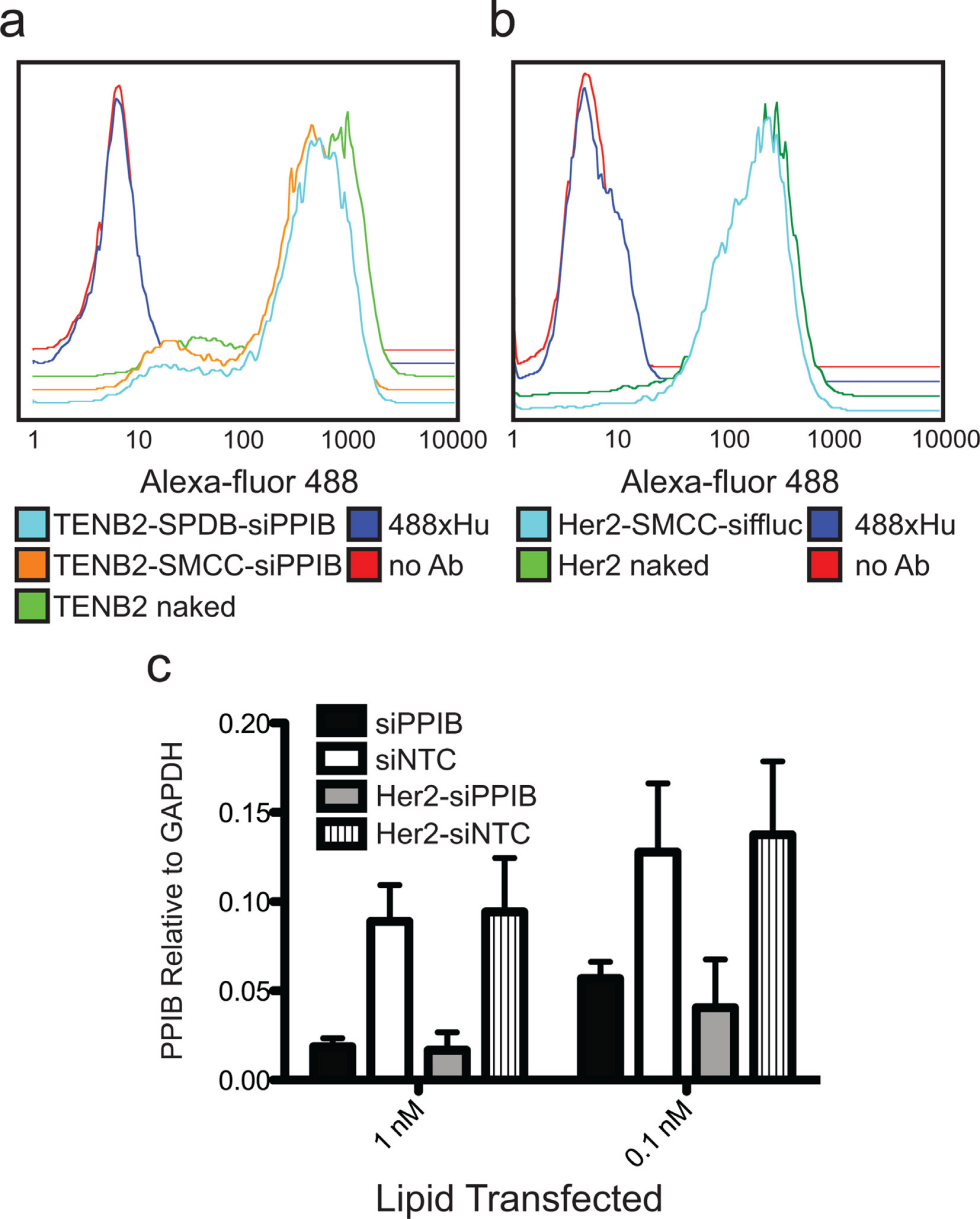
Antibody and siRNA components function normally when coupled in ARCs. (**a**) Flow cytometry comparing binding of naked anti-TENB2 antibody (control) compared to anti-TENB2 ARC (with both SMCC and SPDB linkers, as indicated) to PC3-TENB2-High cells; 488xHu, secondary antibody alone; no Ab, control without primary antibody or ARC added. (**b**) Flow cytometry as in (a) but using PC3-Her2 cells and naked Trastuzumab (anti-Her2) antibody or Trastuzumab ARC. (**c**) PPIB silencing, expressed as an average of PPIB mRNA levels relative to GAPDH mRNA levels, following lipid transfection of free siRNA or siRNA conjugated in Trastuzumab ARCs, as indicated. siPPIB, siRNA targeting PPIB; siNTC, non-targeting control siRNA against firefly luciferase, ffluc; error bars represent standard deviation, *n* = 2 independent experiments.

We first assessed whether covalent coupling of a highly negatively charged macromolecule, the siRNA, affected the ability of the antibody component of ARCs to bind cell surface antigens. Flow cytometry demonstrated that both anti-TENB2 and anti-Her2 ARCs bound to cell surface TENB2 and Her2, respectively, at levels similar to those observed using the naked antibodies (Figure 2a and b), independent of linker choice (Figure 2a). Thus, siRNA coupling did not interfere with antigen binding on the cell surface.

We also examined whether siRNAs (∼15 kD) retained silencing activity when conjugated to antibodies, asking whether covalent attachment to a large protein (∼150 kD) might interfere with silencing activity. To assess siRNA-induced silencing activity inside of cells, we initially sought a method that would directly deliver the conjugates into cells, independent of the receptor-mediated internalization route that we ultimately desired to test. Following reports of lipid transfection of proteins, including antibodies (17), we used lipid transfection to artificially and directly deliver ARCs or siRNAs to the cytoplasm, circumventing receptor internalization. Comparing silencing activities of equimolar concentrations of ARCs or free siRNAs, we found that ARC transfection induced silencing as efficiently as did transfection of the free siRNAs, with ∼50% silencing remaining even at low concentrations (0.1 nM, Figure 2c). Such efficient silencing following ARC transfection using lipids has been extremely reproducible and consistent across our ARC preparations, including tests of multiple distinct antibodies and siRNAs. Thus, we conclude that covalent coupling to an antibody does not impair the ability of delivered siRNAs to mediate RNAi.

### A broad platform of ARCs to assess antibody, siRNA and cellular properties that impact delivery and silencing

The first reports of antibody-mediated siRNA delivery focused narrowly on individual antibody–antigen interactions and only one or a few cell types. We aimed to study ARC-mediated delivery more broadly and to survey how a variety of properties of the ARCs and targeted cells affected silencing. Therefore, we developed an ‘ARC platform’ employing antibodies targeting seven distinct cell surface antigens (Table 1). Reasoning that the route of antigen internalization and intracellular trafficking could affect delivery to the RISC, we surveyed antigens that traffic differently, including three that traffic to the lysosome (TENB2; Steap1, six transmembrane epithelial antigen of the prostate; EtBr, endothelin B receptor), two that initially recycle back to the cell surface (Her2, NaPi2b) and two that internalize slowly (Mesothelin, Muc16) (18–22). To determine whether antigen expression levels affected silencing, we also examined multiple cell lines that express different cell-surface numbers of many of the antigens. Each of the siRNAs used in the ARC platform were products of a rigorous process designed to minimize off-target effects while maintaining highly efficient silencing (>90% following transfection of sub-nanomolar siRNA concentrations). We routinely used a siRNA that targeted PPIB, a housekeeping gene, because efficient PPIB knockdown does not affect viability in the cells and growth conditions examined. As control siRNAs, we used non-targeting siRNA sequences against firefly luciferase or PPIBmm siRNA, containing a two base pair mismatch (mm) at the cleavage site, an alteration that prevents mRNA cleavage.

**Table 1.** List of antigens targeted, cell lines and approximate antigen expression levels, route of bulk antigen internalization and summary of silencing results observed following ARC-mediated delivery and antigen internalization

Antigen	Cell lines/expression levels	Internalization Route	Silencing
Control	PC3-TVA/none	N/A	None
Control	293-WT/none	N/A	None
TENB2	PC3-Tenb2-medium/94 000 copies	Lysosome	+
TENB2	PC3-Tenb2-high/1 700 000 copies	Lysosome	+++
Steap1	Steap1-293/264 000 copies	Slow internalizing	+
Steap1 TENB2	LnCap-Ner-22RV1/very low	Slow internalizing (Steap1), Lysosome (TENB2)	None
Her2	PC3-Her2/100 000 copies	Recycling	None
Her2	MCF7-Her2/600 000 copies	Recycling	None
Her2	SKBR3/2 000 000 copies	Recycling	None
NaPi2b	PC3-NaPi2b/606 000 copies	Recycling	++
NaPi2b	lgrov-1/884 000 copies	Recycling	++
Mesothelin	293-Mesothelin/80 000 copies	Slow internalizing	None
Mesothelin	Capan2/10 000 copies	Slow internalizing	None
MUC6	PC3-Muc16/medium	Slow internalizing	None
MUC6	Capan2/medium	Slow internalizing	None
EtBR	UACC257/34 000 copies	Lysosome	None
EtBR	928-Mel/medium	Lysosome	None

None, ≤10%; +, ≤25%; ++, ≤50%; +++, >50%.

### A screen of the ARC platform reveals that a subset of ARCs mediates siRNA delivery and silencing *in vitro*

**Figure 3.**
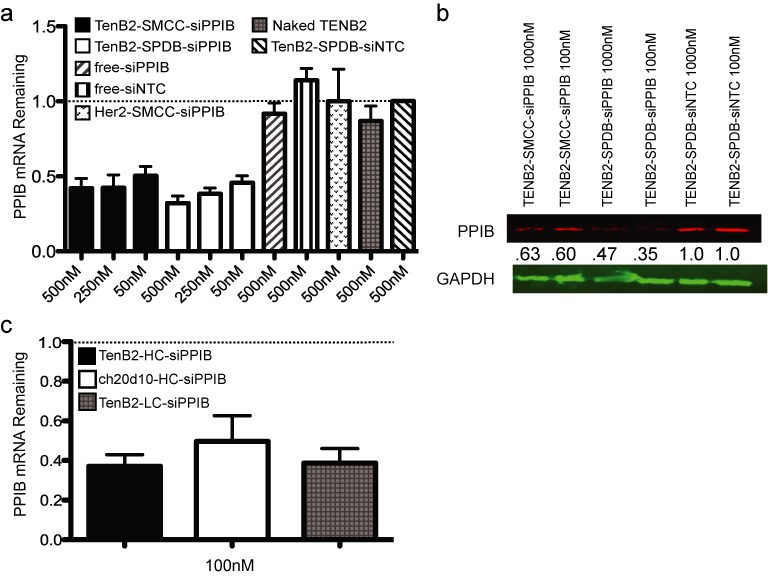
TENB2 ARCs induce silencing *in vitro* independent of antibody and linker format, assessed at the mRNA and protein levels. (**a**) Quantigene expression analysis of PPIB mRNA levels following a 72-h administration to PC3-TENB2-high cells of the indicated ARCs (using non-reducible SMCC or reducible SPDB linkers, as indicated) or, as controls, free antibodies (Naked TENB2) or free siRNAs. Data were normalized to TENB2-siNTC control to allow for comparisons between experiments (dotted line at 1.0); *N* = 10 independent experiments for all samples, except Her2 ARC where *N* = 8. Error bars represent SD. (**b**) Immunoblotting of PPIB protein in PC3-TENB2-high cells following 72 h of treatment with the indicated ARCs. Numbers represent the PPIB protein levels normalized to GAPDH protein levels, expressed relative to the value determined for the TENB2-SPDB-siNTC control. (**c**) Gene expression analysis as in (a) comparing silencing using TENB2 ARC with siRNAs conjugated to the heavy chain (HC), a second TENB2 ARC using a different anti-TENB2 antibody (ch20d1), and a TENB2 ARC with siRNAs conjugated to the light chain (LC). The dotted line represents the value determined for the TENB2-siNTC control (set to 1.0) to which the values for the other groups were normalized (SD shown, *N* = 4; ARCs used the SPDB linker).

We screened the ARC platform to determine whether antibody–antigen receptor-mediated endocytosis could (i) deliver ARCs to the cytoplasm and (ii) support silencing of siRNA-targeted mRNAs. We compared silencing efficiency using seven different antigens, multiple siRNAs and multiple cell lines, typically measuring silencing after 48–72 hours, consistent with timing that is used for typical siRNA transfection experiments (23). The anti-TENB2 ARC consistently showed the most efficient silencing of targeted mRNAs, although receptor internalization did not induce silencing as strongly as observed following lipid transfection (compare Figures 3a and 2c). Addition of the anti-TENB2 ARC reduced mRNA levels by ∼50% at 10–50 nM of ARC to 70% at 500 nM in PC3 cells engineered to express high levels of TENB2 (Figure 3a and Supplementary Figure S2). We typically observed that 10 nM of ARC-induced near-maximal silencing (Supplementary Figure S2) that increased only slightly with increasing ARC concentrations (compare silencing levels in Figure 3a, b and Supplementary Figure S2), suggesting that above 10–50 nM, ARC concentration was not limiting for silencing. As controls, mixtures of free siRNA and free antibody were tested, and both failed to induce silencing, as did control anti-TENB2 ARCs carrying a non-targeting siRNA; thus, silencing depended on the covalent coupling of the targeting siRNA to the antibody (Figure 3a). Immunoblotting confirmed silencing, revealing decreases in PPIB protein levels that correlated with decreases in mRNA levels (Figure 3b). Silencing correlated with receptor expression, as reduced levels of expression resulted in diminished levels of silencing (Table 1, compare rows 3 and 4). Finally, 5′-RACE assays revealed that mRNA cleavage occurred precisely at the predicted position, confirming knockdown mediated via a *bona fide* RNAi mechanism (Supplementary Figure S3) (24).

We performed numerous additional control experiments and examined the consequences of altering the siRNA, antibody, and linker components. In addition to PPIB mRNA, other mRNAs (MAP2K2, UBB, luciferase, PLK1 and HDAC1) were targeted with similar efficiencies when we used anti-TENB2 ARCs carrying the cognate siRNAs (data not shown), demonstrating that ARC-induced silencing could be generally applied to multiple mRNA targets. Importantly, a control ARC with an antibody targeting the Her2 antigen, which is not expressed in PC3-TENB2 cells, did not induce silencing in PC3-TENB2 cells; similarly, the anti-TENB2 ARC did not induce silencing in cell lines not expressing TENB2, including the parental PC3 line lacking the TENB2 transgene (Figure 3a, Supplementary Figures S4A and S4B, and data not shown). Switching to a second anti-TENB2-HC THIOMAB supported silencing in cells expressing the TENB2 antigen (Figure 3c), further supporting the notion that ARC mediated silencing depends on antibody–antigen targeting while further revealing that different epitopes on the targeted antigen can be used. Finally, both the non-reducible (SMCC) and reducible (SPDB) linkers supported silencing (Figure 3a and b, Supplementary Figures S4A and S4D), indicating that both stable and reducible linkers function in the ARC format and, therefore, that linker reduction is not needed to liberate the siRNA for silencing (see section ‘Discussion’ for hypotheses on mechanism). Finally, moving the linker position from the antibody heavy chain to light chain position (V205C) did not alter silencing (Figure 3c), demonstrating that ARC mediated silencing tolerates changes in linker location.

**Figure 4.**
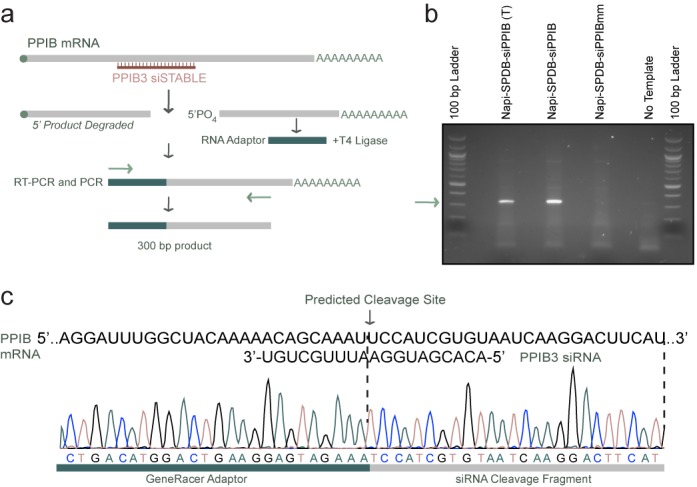
5′ RACE assay demonstrates anti-NaPi2b-ARCs induce silencing via an RNAi mediated mechanism. (**a**) Schematic of the 5′ RACE assay used to determine the cleavage position on PPIB mRNA. (**b**) Agarose (3%) gel electrophoresis analysis of RT-PCR products using RNA isolated from PC3-NaPi2b cells treated with 100 nM of the indicated ARCs (SPDB linkers) for 72 h. The sample designated with (T) is a positive control that used lipid transfection of the ARC, rather than antigen internalization, to deliver the ARC and induce RNAi. Only use of the anti-NaPi-SPDB-siPPIB ARCs generated the 300 bp product indicative of accurate RNAi-mediated cleavage. (**c**) DNA sequencing results of the RT-PCR product using RNA isolated from cells treated with the anti-NaPi-SPDB-siPPIB ARC. Sequencing reads mapped the cleavage site to the indicated position (nucleotide 447 in the PPIB mRNA sequence), the site predicted from a true RNAi cleavage mechanism.

In addition to anti-TENB2 ARCs, anti-NaPi2b ARCs also induced silencing. As with anti-TENB2 ARCs, anti-NaPi2b ARC-induced silencing depended on the siRNA sequence and the correct antibody–antigen interaction (Supplementary Figures S4A and S4B). We confirmed silencing at the mRNA (Supplementary Figure S4A) and protein levels (Supplementary Figure S4B), although silencing appeared more modest when measured by immunoblotting, perhaps reflecting longer turnover time for this multipass protein compared to the mRNA. Again, we observed similar silencing efficiencies using either the non-reducible or reducible linkers (Supplementary Figure S4A). Silencing reflected a *bona fide* RNAi mechanism because 5′-RACE assays revealed precise mRNA cleavage at the expected position (Figure 4). These results demonstrate that ARC mediated silencing (to a maximum of ∼75%) extends to at least two antigens, TENB2 and NaPi2b. Furthermore, these antigens internalize and traffic differently (to lysosomes and via recycling endosomes, respectively; see Table 1), suggesting that silencing can be achieved through at least two different routes of antigen internalization.

The screen also revealed limitations to the approach, however, given that ARCs targeting the five additional antigens induced weak or no silencing. Specifically, anti-Steap1-siPPIB ARCs induced PPIB silencing in 293 cells engineered to express Steap1 (Steap1-293 cells), but this silencing, although reproducible, did not exceed a 40% reduction in PPIB mRNA levels, even at high ARC concentrations (Supplementary Figure S4D). The remaining antigens, including Her2, did not induce silencing. We focused on analysing this negative result using anti-Her2 ARCs because we possessed a deep arsenal of reagents related to Her2, and anti-Her2 delivery of siRNAs had been previously reported (9,11,25). Using the rigorous battery of control tests described above for anti-TENB2 ARCs to define *bona fide* silencing, we could not detect silencing using anti-Her2 based ARCs. Given that our results contrast with previous reports using anti-Her2 antibodies and aptamers to induce RNAi via Her2 internalization (9,11,26,27), we thoroughly tested multiple versions of Her2 ARCs—including antibodies against different epitopes, bi-specific Her2-EGF antibodies, full-length and Fab antibody formats, linker positions, linker chemistry and siRNA targets. We also examined a variety of growth conditions using multiple cell lines, including three human cancer lines that express high levels of either endogenous Her2 (SKBR3, ‘3+’ expression; see Supplementary Figure S4C) or stably transfected Her2 (in MCF7 and PC3 cells; data not shown). The consistent lack of anti-Her2 induced silencing cannot be attributed to a general disruption of anti-Her2 or siRNA activities when the antibody and siRNA components are covalently coupled in an ARC because: (i) anti-Her2-siPPIB ARCs retained the ability to bind Her2 antigen-presenting cells and (ii) the same ARCs mediated silencing when delivered using lipid transfection (Figure 2 and Supplementary Figure S4C).

### ARC egress from endosomes may limit silencing efficiency

**Figure 5.**
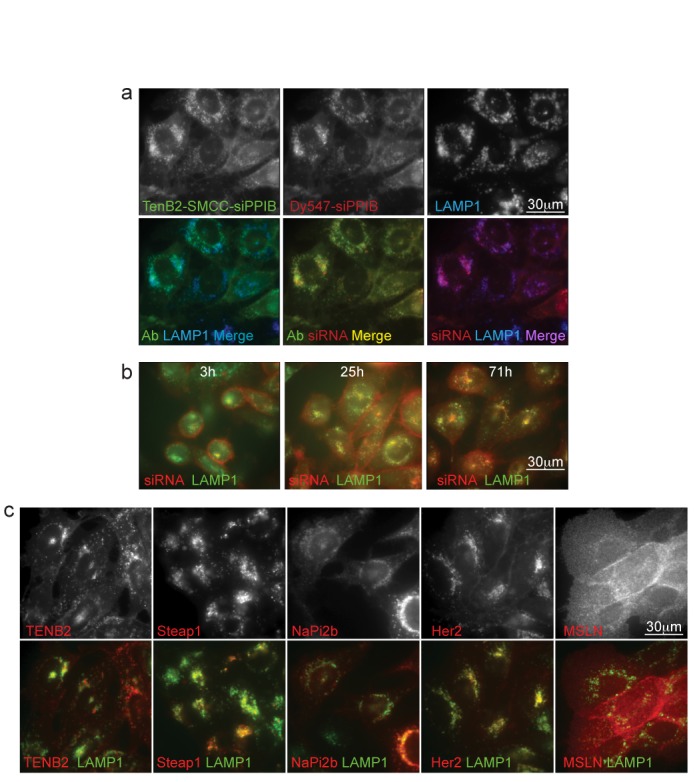
The antibody and siRNA components of both silencing-active and silencing-inactive ARCs traffic to lysosomes. (**a**) Anti-TENB2 ARCs deliver anti-TENB2 and the siRNA payload to lysosomes. PC3-TENB2-high cells were treated for 40 h with anti-TENB2-SMCC-siPPIB ARCs in the presence of lysosomal protease inhibitors. Antibody (green) and late endosome-lysosome (LAMP1 marker, blue) localizations were visualized using IF, and the siRNA (red) was tracked using a Dy4547 label on the 5′ end of the siRNA sense strand. Top row, localization patterns for each individual component; bottom row, merged patterns, as indicated. All three markers show overlapping localization. (**b**) Time course of ARC lysosomal delivery. The experiment was performed as in (a) with images captured at the indicated times. LAMP1, green; siRNAs, red. (**c**) Comparison of antibody internalization. The experiment was performed as in (a), except using antibodies targeting the indicated antigens and the cognate antigen-expressing cell lines, with continuous uptake for ≥20 h. Anti-Mesothelin (MSLN), which is internalized very slowly, served as a control for surface membrane localization after 19 h. Top row, antibody localization alone; bottom row, antibody (red) and LAMP1 (green) localizations merged.

We hypothesized that differences in antigen internalization and ARC trafficking within cells could explain why some ARCs induce silencing but others do not. Therefore, we used immunofluorescence (IF) microscopy to track delivery of the bulk populations of both the antibody and siRNA components of ARCs, initially focusing on the anti-TENB2 ARC, the ARC from our platform that most effectively induced RNAi. Under the same conditions (continuous uptake) employed during our silencing experiments and at a time (40 h) when silencing could be detected (Supplementary Figure S2), we found that (i) the antibody and labeled siRNA co-localized and (ii) this co-localization overlapped with the late endosomal and lysosomal marker LAMP1 (Figure 5A). Analysing anti-TENB2 ARC uptake over a time course revealed that the bulk of the ARC population appears bound at the cell surface at 3 h, maintains some surface localization while also appearing in lysosomes at 25 h, and appears largely lysosomal by 71 h (Figure 5b). These initial studies relied on labeling of only one siRNA strand to track siRNA localization and followed the entire population during continuous uptake rather than during a ‘chase’ to the destination compartment. To track both strands, we generated a dual labeled ARC, containing Dy547 at the 3′ position of the antisense strand and Dy647 at the 5′ position of the sense strand. IF microscopy revealed patterns indistinguishable from those observed by tracking only one strand; namely, the bulk population of both strands co-localized and trafficked to lysosomes (Supplementary Figure S5) over a 23–64 h time course (Supplementary Figure S6), even after a pulse-chase to monitor trafficking to the destination compartment. IF microscopy also did not detect siRNAs co-localizing with the P-body marker Dcp1a (Supplementary Figure S6). Thus, at least in the bulk ARC population that can be tracked by IF methods and remains stable and detectable, the antibody and siRNA components traffic together to the lysosome and are not found in P-bodies during the period in which silencing reaches a maximum (Supplementary Figure S2). We note that although lipid transfection of free siRNA induces highly efficient silencing, the IF methods also do not detect strand separation or P-body localization when tracking such transfected free siRNA (Supplementary Figure S6).

The THIOMABs investigated in this study bind cell-surface antigens and internalize via different routes (slow/non internalizing, recycling, lysosomal). Control pulse-chase experiments revealed that the ARCs use the same initial (<4 h after internalization initiates) trafficking routes as the cognate, naked antibodies (data not shown). To determine whether there was a correlation between ARC internalization routes and silencing activity, we used IF to track the intracellular locations of four different antibodies used in our ARC platform. We used the mesothelin antigen as a control for very slow internalization (18), to demonstrate cell surface localization (Figure 5c). Regardless of the ascribed initial internalization routes, the bulk of the four antibodies co-localized with LAMP1 (Figure 5c). Thus, trafficking appeared similar for all antibodies, whether the cognate ARC induced silencing (e.g. anti-TENB2) or failed to induce silencing (e.g. anti-Her2). The silencing consistently observed using anti-TENB2 ARCs implies that at least a small fraction of the internalized siRNAs must enter the RISC and separate strands. IF microscopy, which tracks bulk populations, appears unable to distinguish differences in the intracellular trafficking and RISC delivery routes that we presume the ‘silencing’ and ‘non-silencing’ ARCs must employ.

**Figure 6.**
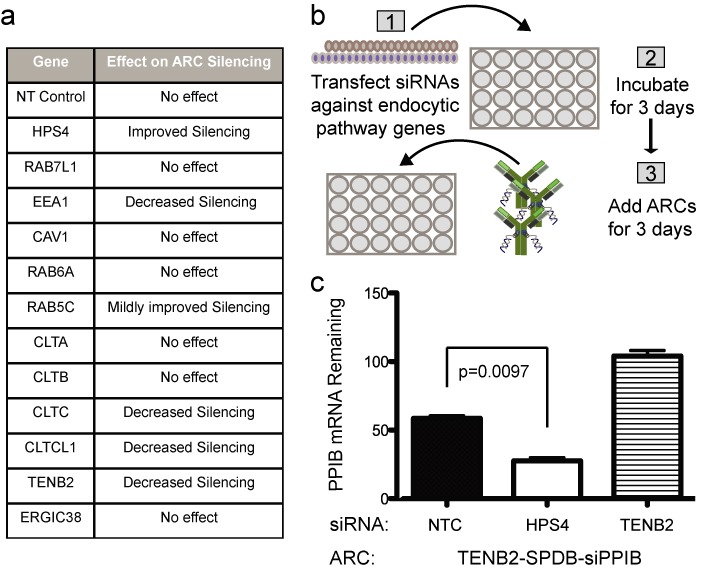
siRNA screen of endocytic pathway components reveals that HSP4 inhibition improves ARC-mediated silencing efficiency. (**a**) List of genes targeted in the candidate-based siRNA screen and the observed phenotypes following knock down. (**b**) Schematic of the experimental approach. We transfected TENB2-PC3-High cells with siRNAs against endocytic pathway genes and incubated for 3 days. We then added anti-TENB2-SPDB-siPPIB ARCs to the cells and assayed ARC-mediated silencing of PPIB mRNA after 3 additional days. (**c**) ARC-mediated silencing of PPIB mRNA after initial transfection with siRNA targeting HPS4, siRNA targeting TENB2 (control, to reduce levels of the antibody target) or non-targeting control (NTC) siRNA (SD shown, *N* = 4).

Based on our observations that ARCs enter cells via the endocytic pathway as well as previous reports linking small RNA-silencing to endosome trafficking, we performed a candidate-based screen (Figure 6a) to identify components of the endocytic pathway that could affect the efficiency of ARC-mediated silencing. Our strategy used (i) lipid transfection of siRNAs to knock down components of the endocytic pathway and (ii) assays of whether such knockdown affected subsequent ARC-mediated silencing (Figure 6b). Intriguingly, knockdown of one endocytic component, HPS4, nearly doubled the efficiency of silencing induced using anti-TENB2-siPPIB ARCs (Figure 6c). Thus, HPS4 appears to negatively impact ARC silencing, consistent with the previous finding that HPS4 disruption enhances siRNA and miRNA silencing (28). Although HPS4 knockdown improved the activity of ‘silencing-active’ ARCs, such as anti-TENB2 and anti-NaPi2b ARCs, it did not impart silencing to inactive ARCs, such as those based on anti-HER2. These data indicate that components of the endosomal and lysosomal pathways may be critical to support functional ARC-mediated silencing.

### TENB2 ARC delivery to tumors *in vivo*

**Figure 7.**
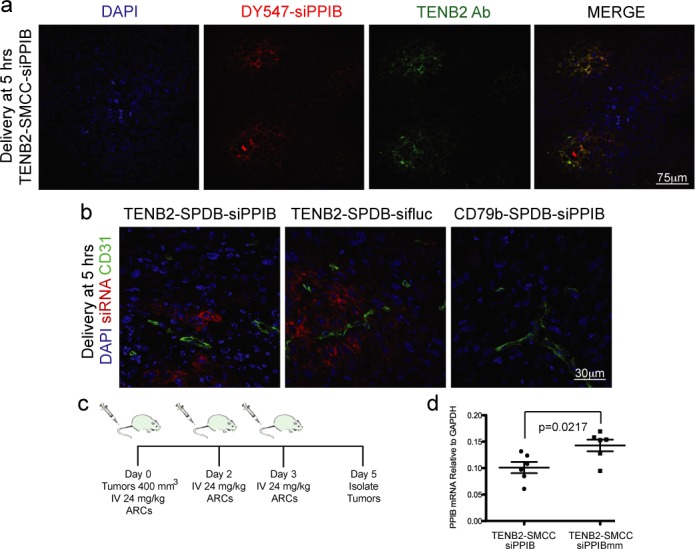
ARCs are delivered to tumors *in vivo* and mediate silencing. (**a**) TENB2 antibody and siRNAs (Dy-547 labeled) are co-delivered to tumor cells within 5 h after intravenous injection of ARCs. (**b**) Visualization of siRNAs (Dy-547 labeled) and the endothelial cell maker CD31 in tumor sections reveals siRNA delivery to tumor cells near the vasculature in a manner that depends on the targeted antibody–antigen interaction. (**c**) Schematic of *in vivo* silencing study. We dosed mice bearing tumors with a volume of ∼400 mm^3^ three times with 24 mg/kg (total ARC mass in mg per body weight in kg, equivalent to ∼20.6 mg/kg antibody plus 3.4 mg/kg siRNA) of ARCs and harvested tumors on day 5. (**d**) Assessment of TENB2 ARC induced silencing *in vivo* (in EpCam+ cells isolated from near the CD31+ tumor vasculature); PPIB mRNA levels, normalized to GAPDH mRNA levels, are shown for each animal (*N* = 6 for each group, SD shown, Student's *t*-test: *P* = 0.0217).

The demonstration of competent *in vitro* silencing using anti-TENB2 ARCs prompted us to investigate whether ARCs could deliver siRNAs to tumors *in vivo*. We performed delivery studies in *nu/nu* mice harboring 200 mm^3^ tumors generated from xenografts of PC3-TENB2-high cells. We intravenously injected tumor-bearing mice with a single dose of anti-TENB2-siPPIB ARC. As a control for siRNA sequence, we used anti-TENB2-siPPIBmm and, as a control for antibody targeting of the tumor cells, we used anti-CD79b-siPPIB (these tumor cells do not express CD79b). The anti-TENB2-siPPIB ARC quickly (by 5 h) localized to the tumor, particularly near the tumor vasculature, and the labeled siRNAs and anti-TENB2 antibodies co-localized, indicating that ARCs remained intact within the tumor (Figure 7a and b). Importantly, we did not detect the control anti-CD79b ARC in or near the tumor (Figure 7b), indicating that the localization of the anti-TENB2 ARC components depended on the proper antibody–antigen interaction.

We next determined whether anti-TENB2 ARCs induced silencing in xenografted tumors. We dosed tumor-bearing mice with ARCs (Figure 6c) and harvested tumors after 5 days. Given the results in Figure 6b, we focused our analysis of PPIB mRNA levels to EpCAM-positive tumor cells near the vasculature to enrich for cells in which ARCs had been delivered. During this short period of ARC treatment, we found that anti-TENB2-siPPIB ARCs silenced PPIB mRNA expression by ∼33% (*P* = 0.02) relative to control ARCs carrying the mm siRNA control (Figure 6c, *N* = 6, Student's *t*-test).

## DISCUSSION

Previous studies of RNAi delivery using antibody–siRNA complexes have relied on a limited set of cell lines and antibodies, and complex formation has been driven by charge–charge interactions, yielding aggregates unsuitable to the rigorous demands of therapeutic manufacturing. To thoroughly test antibody-mediated delivery and whether such technology could be industrialized for clinical use, we analyzed ARCs against a panel of seven distinct antigens, using clinical grade THIOMABs. Anticipating that the route of antigen internalization might affect silencing, we selected antigens that use multiple trafficking pathways. We also varied numerous other parameters, examining: (i) multiple cell lines expressing various levels of the antigens, (ii) reducible versus non-reducible linkers, (iii) different linker positions, (iv) different antibodies against the same antigen and (v) full-length antibodies versus Fab fragments.

The use of THIOMABs represents a significant improvement in conjugation methods. Developed for clinical use in ADCs (antibody–drug conjugates, for cytotoxin delivery to tumor cells), THIOMABs have been engineered to include two free cysteine residues (at positions that do not disrupt antibody function) as anchors for covalent coupling to linkers. Our purified ARC preparations were comprised of a well-defined mix of THIOMABs conjugated to one or two siRNAs, at typical ratio of 1:2, respectively. Our conjugates contrast with previous antibody fusions to positively charged peptides, which electrostatically bind the negatively charged siRNAs and likely generate populations of aggregates with poorly defined and varied antibody–siRNA compositions. THIOMABs have enabled us to generate pure, well-defined monomers using a covalent coupling technology that was designed at the onset for clinical use and drug manufacturing.

Characterization of the antibodies and siRNAs in the ARC context indicated that both components retained activity. Using multiple antibodies and siRNAs, we found that ARCs induced highly efficient, reproducible silencing when directly delivered using lipid transfection. Although the mechanism of RISC engagement is unknown, we speculate that the linkers may provide spacing that allows for siRNA incorporation into the RISC, even with the conjugate intact. Given that the siRNAs are conjugated via the sense strand, unwinding of the siRNA duplex and RISC incorporation of the single antisense strand would be predicted to liberate the antisense strand from the ARC. Alternatively, the antibody and siRNA may separate between transfection and RISC engagement. Such separation, if it occurs, does not appear to be due to linker reduction because both the reducible and non-reducible linkers support silencing. However, antibody or linker degradation could liberate the siRNA drug and, indeed, such a mechanism has been proposed to explain how small molecule cytotoxins are liberated in antibody–drug conjugates (29).

One highlight from screening our ARC panel was the identification of two ARCs, based on anti-TENB2 and anti-NaPi2b, that consistently induced silencing, including at modest concentrations. A battery of controls conclusively determined that silencing reflected ARC internalization and a true RNAi mechanism. Silencing absolutely depended on matched receptor and antibody pairs, antigen expression, and an active siRNA sequence, and it tolerated a variety of ARC modifications, including different antibodies against the targeted antigen, antibody formats and linker positions. Notably, both reducible and non-reducible linkers supported silencing; thus, linker reduction to liberate the siRNA from the antibody, as might be expected to occur under the reducing conditions in an endosomal compartment, is not essential for the siRNA to engage the RISC. Our IF microscopy studies also indicated that the bulk of the siRNA and antibody populations co-localize within cells, consistent with the two molecules remaining together. Of course, siRNA could be liberated from the antibody through other mechanisms, such as antibody or linker degradation, and co-localization does not assess covalent attachment.

Although these two ARCs reproducibly induced RNAi when delivered through antigen internalization, silencing efficiency was moderate (50–75% reduction in targeted mRNA levels) compared to that observed following lipid transfection (>95%). Fifty to seventy percent silencing appeared to represent a ceiling because increasing ARC concentrations, even to 500 nM, failed to significantly improve silencing. Thus, lipid transfection overcomes limitations inherent to delivery via antigen internalization. Furthermore, silencing efficiency declined when we targeted cells expressing fewer antigen molecules (∼10^5^ cell surface antigens) compared to cells expressing greater numbers (∼10^6^) (Table 1), pointing to antigen expression as a limiting factor.

A goal for ARC technology *in vivo* was not only to deliver siRNAs into cells but also to enhance delivery to the surface of the targeted cells and targeted tissue, while avoiding accumulation elsewhere. We found that ARCs successfully delivered siRNA to tumor cells *in vivo* in a targeted manner. However, delivery appeared localized to the tumor cells flanking the vasculature, perhaps reflecting limitations to ARC penetration into poorly vascularized tumors. RNAi targeting *in vivo* has been previously reported using transferrin to deliver nanoparticles carrying siRNA cargo. In this case, ‘targeting’ refers to enhanced cellular uptake and silencing, rather than selective accumulation in the targeted tissue. Indeed, transferrin targeting did not affect biodistribution, as both targeted and non-targeted particles accumulated to a similar extent in the tumor microenvironment (due to the enhanced permeability and retention effect) as well as in the liver and kidneys.

We detected RNAi in tumor cells following systemic ARC administration *in vivo*, although silencing was limited to an approximate 33% reduction in the mRNA target in the tumor cells near the vasculature. On one hand, this demonstration of ARC mediated silencing provides a foundation for generating an antibody-based technology that enables targeted, systemic delivery for RNAi in tumors, a major challenge for the field. On the other hand, making this technology generally applicable requires overcoming multiple limitations. In addition to the challenges of ARC entrapment in endosomal compartments, pharmacokinetic studies in mice suggest that ARCs are cleared faster than the corresponding naked antibodies (data not shown, Figure 5b). We hypothesize that ARC modifications—for example, adding molecular masks to protect the siRNAs and shield negative charge (employing a rationale similar to that behind dynamic polyconjugates)—might enhance ARC stability in circulation and facilitate endosomal release (30,31).

In contrast to the anti-TENB2 and anti-NaPi2b ARCs, ARCs targeting the other five antigens supported little or no silencing activity. This inactive class included ARCs based on anti-Her2, notable given previous reports that anti-Her2–siRNA complexes mediate siRNA delivery and silencing (9,11,25) and that Her2 is a highly active target for antibody–drug conjugates. Our observation that anti-Her2 ARCs did not induce silencing held under a battery of experimental formats, including using cell lines that express very high surface levels of Her2. To determine whether the use of positively charged polypeptides could explain the previous successful reports of anti-Her2-based siRNA delivery, we generated our own anti-Her2–protamine–siRNA complexes; however, these complexes also did not induce silencing (data not shown). We are left to conclude that, independent of protamine versus direct conjugation methods, anti-Her2 antibodies do not efficiently support antibody-mediated silencing, at least in the cell lines and conditions employed in our studies.

Our ARC platform enabled us to query whether the internalization route of the targeted antigens impacted silencing efficiency. Relying on fluorescent microscopic imaging techniques, we discovered that the siRNA and antibody components traffic together within the cell, with the bulk ARC population ultimately delivered to lysosomes. Lysosomal trafficking occurred for all of the ARCs that are internalized, independent of the bulk trafficking routes previously categorized for the antigens. At least one ARC from each trafficking category supported some silencing, and thus there was no correlation between the bulk trafficking route and silencing. For example, Her2 and NaPi2b have been characterized as transmembrane proteins that cells internalize and recycle, and our IF microscopy experiments confirmed that the bulk populations of ARC targeting these antigens trafficked similarly. Nevertheless, anti-NaPi2b ARCs induced silencing whereas anti-Her2 ARCs did not, suggesting that there must be a silencing competent fraction of anti-NaPi2b ARCs that trafficks differently from the bulk population, escaping lysosomal compartmentalization and degradation to reach the RISC intact. Neither P-body localization nor strand separation were observed when using IF methods to track ARC-delivered siRNA during a time course in which active silencing was detected; similar results were observed using transfected free siRNAs under conditions of highly efficient silencing. Silencing may occur in sub-microscopic structures (32), and estimates suggest that very low levels of cytoplasmic siRNA—approximately a few hundred to a few thousand siRNAs per cell—can support silencing. Thus, ARC-induced silencing may reflect successful delivery of only a small fraction of the internalized population, a fraction that would be missed by microscopic studies following bulk flow or pulse-chase.

Given our trafficking studies, we reasoned that manipulation of endocytic pathways is a key area for future improvements. Previous reports have linked silencing to endocytosis (28,33), and *Drosophila* scavenger receptors take up dsRNA and deliver it to the RISC via the endocytic pathway (34,35). Through a focused screen of endocytic components, we discovered that HPS4 levels limit ARC silencing efficiency. Mutations in HPS4 can cause Hermansky–Pudlak syndrome, characterized by perturbed biogenesis of lysosome-related organelles (36). Our result extends previous findings showing that HPS4 negatively regulates both miRNA and shRNA silencing, consistent with the notion that HPS4 may inhibit siRNA egress from the late endocytic pathway to the RISC (28,37,38). Future studies will aim to elucidate the mechanism of delivery to the RISC, with the hope that such insights will illuminate basic biology of the trafficking pathways as well as means for improving silencing efficiency.

Our overarching goal was to assess whether antibody–siRNA conjugates could be meaningfully considered to meet the rigorous demands of drug development and targeted siRNA delivery to tumors. Our broad ‘ARC platform’ advances studies of antibody delivery of siRNAs by: (i) providing a panel of antibody–antigen pairs and cell lines to test multiple parameters predicted to impact delivery and silencing and (ii) employing methods that generate high-quality monomeric conjugates—using clinical-grade THIOMABs and precise siRNA–antibody conjugation—that could be realistically considered for drug manufacturing. We discovered that both components of ARCs retain activity, and two of the ARCs supported moderately strong silencing. However, the highest silencing efficiencies were observed only with high antigen expression, and other ARCs supported only weak or no silencing. Thus, improvements must be made to generally enable the method for effective and reliable silencing, particularly *in vivo*. All of the internalizing ARCs delivered siRNA into cells in a targeted manner, and so it seems the challenge to silencing rests in delivering the siRNA not just into cells but also out of endosomal compartments, to the productive intracellular locale for RISC engagement. Continued elucidation of ARC delivery mechanisms will likely illuminate ways to modify the conjugates to facilitate endosomal egress and access to the RISC.

## SUPPLEMENTARY DATA

Supplementary Data are available at NAR Online.

SUPPLEMENTARY DATA
