# Supplementary material for: Systematic evaluation of antibody-mediated siRNA delivery using an industrial platform of THIOMAB–siRNA conjugates
Source: Nucleic Acids Res. 2014 Dec 30;43(2):1189–203. doi: 10.1093/nar/gku1362 (PMC4333408; doi:10.1093/nar/gku1362)
Supplement: SUPPLEMENTARY DATA [file supp_43_2_1189__index.html]

Systematic evaluation of antibody-mediated siRNA delivery using an industrial platform of THIOMAB–siRNA conjugates — Systematic evaluation of antibody-mediated siRNA delivery using an industrial platform of THIOMAB–siRNA conjugates — SUPPLEMENTARY DATA 

# Systematic evaluation of antibody-mediated siRNA delivery using an industrial platform of THIOMAB–siRNA conjugates

## SUPPLEMENTARY DATA

**Files in this Data Supplement:**

- SUPPLEMENTARY DATA
