# Supplementary material for: Systematic evaluation of antibody-mediated siRNA delivery using an industrial platform of THIOMAB–siRNA conjugates
Source: Nucleic Acids Res. 2014 Dec 30;43(2):1189–203. doi: 10.1093/nar/gku1362 (PMC4333408; doi:10.1093/nar/gku1362)
Supplement: SUPPLEMENTARY DATA [file supp_gku1362_nar-00889-y-2014-File010.pdf]

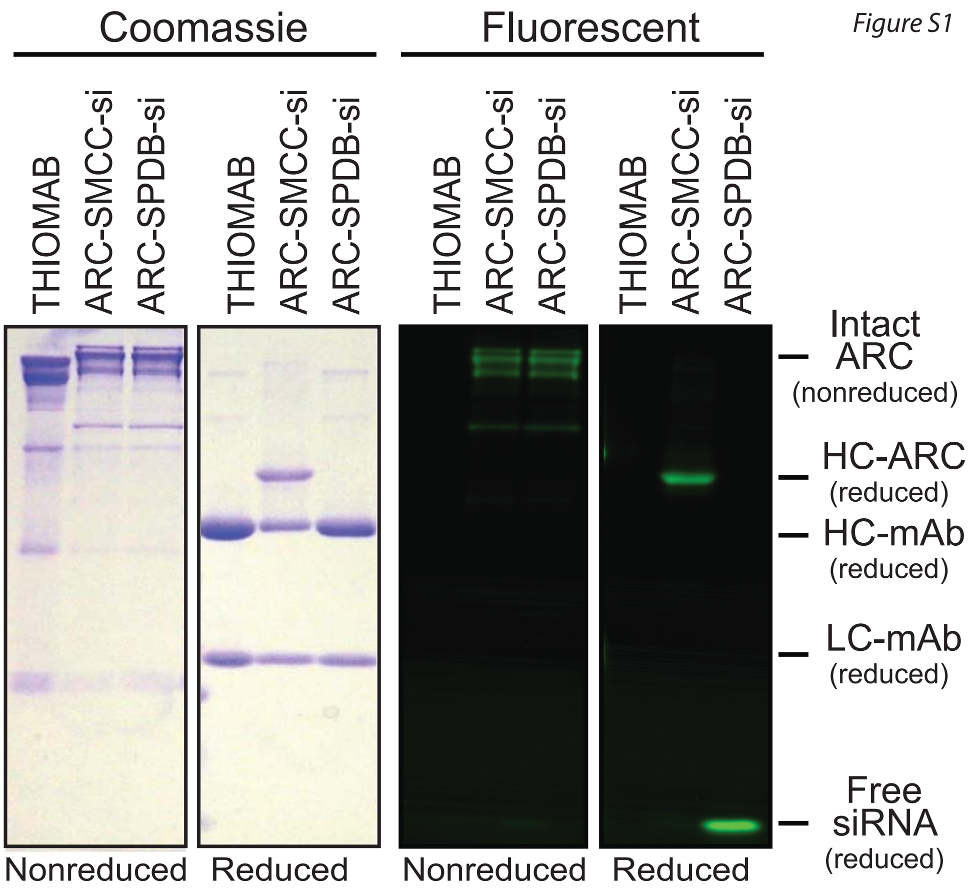
Figure S1
Coomassie
Fluorescent
THIOMAB
ARC-SMCC-si
ARC-SPDB-si
Intact ARC (nonreduced)
HC-ARC (reduced)
HC-mAb (reduced)
LC-mAb (reduced)
Free siRNA (reduced)
Nonreduced
Reduced

Figure S1. ARC preparations are pure and siRNAs are covalently attached to antibodies. Coomassie and fluorescent SDS-PAGE analyses of the ARCs and free THIOMAB controls demonstrate that siRNAs are covalently conjugated onto the heavy chains of THIOMABs (Dy547-labeled siffluc siRNAs and an anti-Her2 THIOMAB are shown). Reducing conditions disrupt the antibody disulphide bonds as well as the SPDB linker between the siRNA and antibody; in contrast, the non-reducible SMCC linker remains intact. THIOMAB, THIOMAB alone (no siRNA conjugated); ARC-SMCC-si, ARC using non-reducible SMCC linker; ARC-SPDB, ARC using reducible SPDB linker; HC-mAb, antibody heavy chain (no siRNA conjugated); LC-mAb, antibody light chain (no siRNA conjugated); HC-ARC, antibody heavy chain conjugated to siRNA.

Figure S2

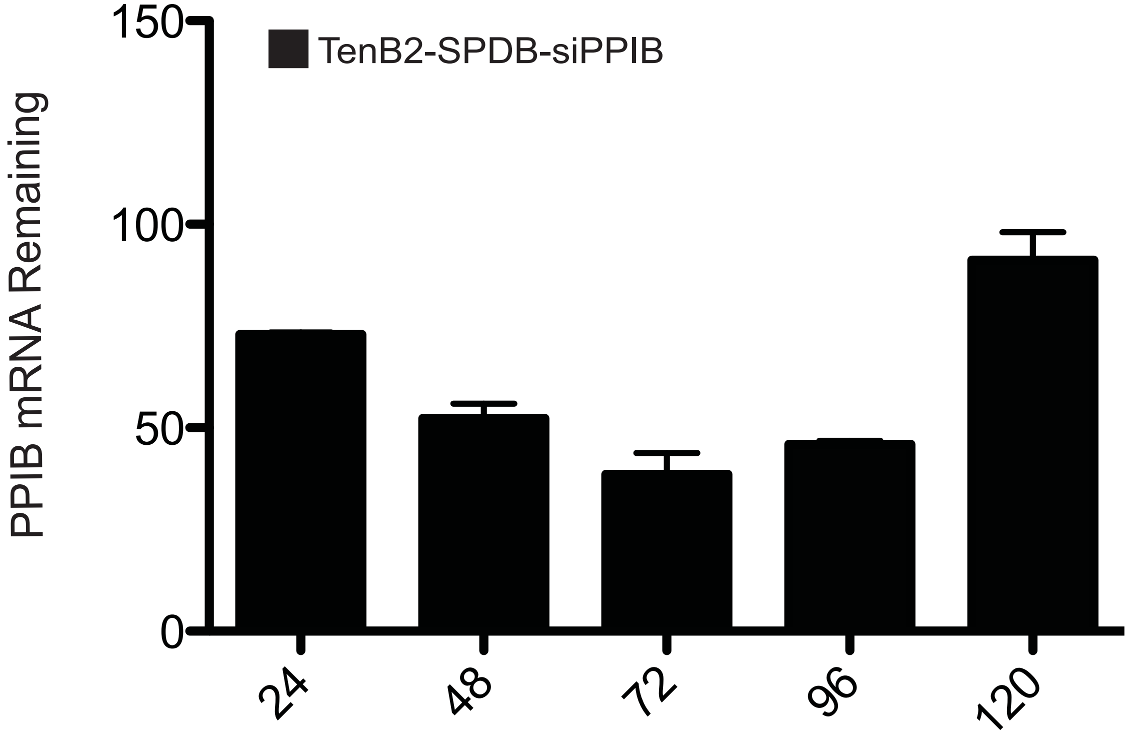

TenB2-SPDB-siPPIB
PPIB mRNA Remaining
150
100
50
0
24
48
72
96
120

Figure S2. Time-course of ARC silencing. Quantigene gene expression analysis of PPIB3 mRNA (relative to GAPH, as in Figure 3) following treatment with 10 nM of TENB2-SPDB-siPPIB ARC for the indicated hours.

Figure S3

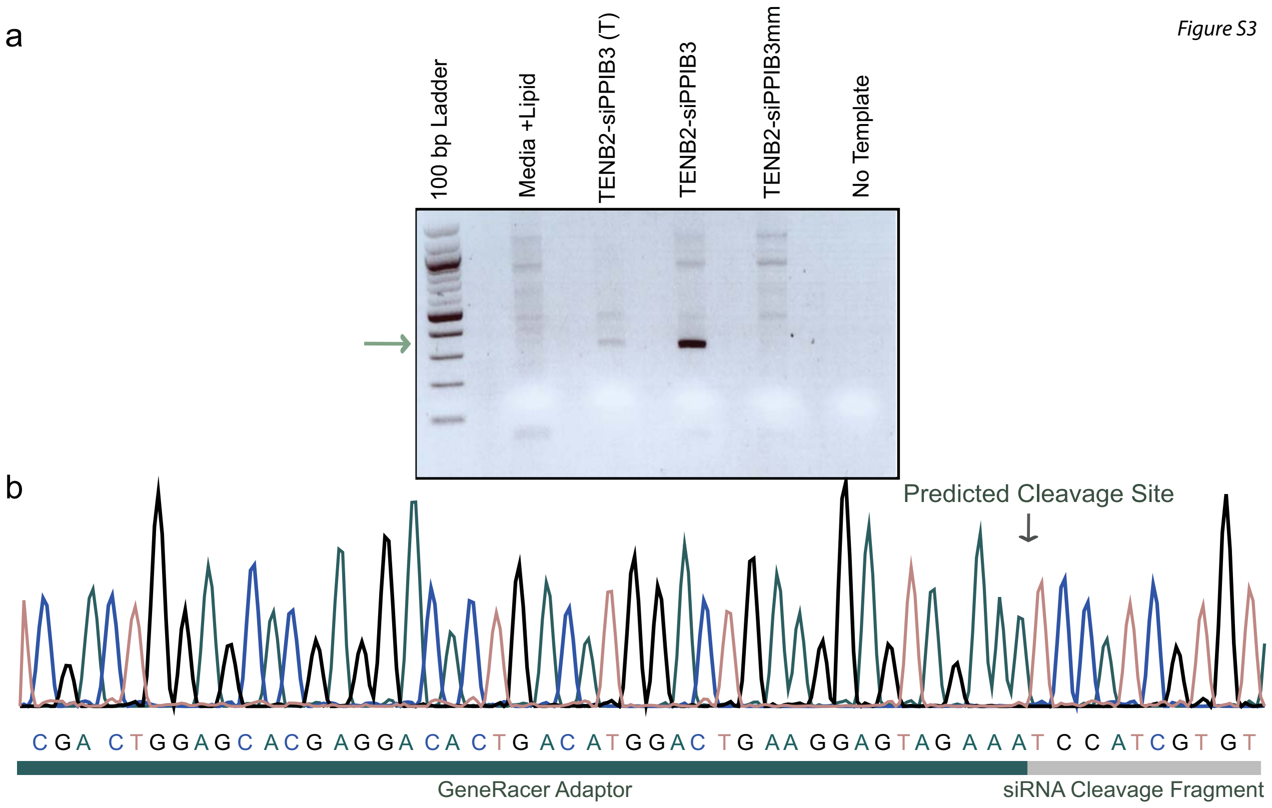
a
100 bp Ladder
Media +Lipid
TENB2-siPPIB3 (T)
TENB2-siPPIB3
TENB2-siPPIB3mm
No Template
b
Predicted Cleavage Site
CGA CTGGAGCACGAGGACACTGACATGGACTGAAGGAGTAGAAATCCATCGTGT
GeneRacer Adaptor
siRNA Cleavage Fragment

Figure S3. 5' RACE assay demonstrates TENB2-ARCs induce silencing via an RNAi mediated mechanism. (a) Agarose (3%) gel electrophoresis of RT-PCR products using RNA isolated from PC3-TENB2 high cells treated with 100 nM of the indicated ARCs (SPDB linkers) for 72 hours. The sample designated with (T) is a positive control that used lipid transfection of the ARC, rather than antigen internalization, for delivery. (b) DNA sequencing results of the RT-PCR product using RNA isolated from cells treated with the TENB2-SPDB-siPPIB ARC. Sequencing reads mapped the cleavage site to the indicated position (nucleotide 447 in the PPIB mRNA sequence), the cleavage site predicted for a true RNAi cleavage mechanism.

Figure S4

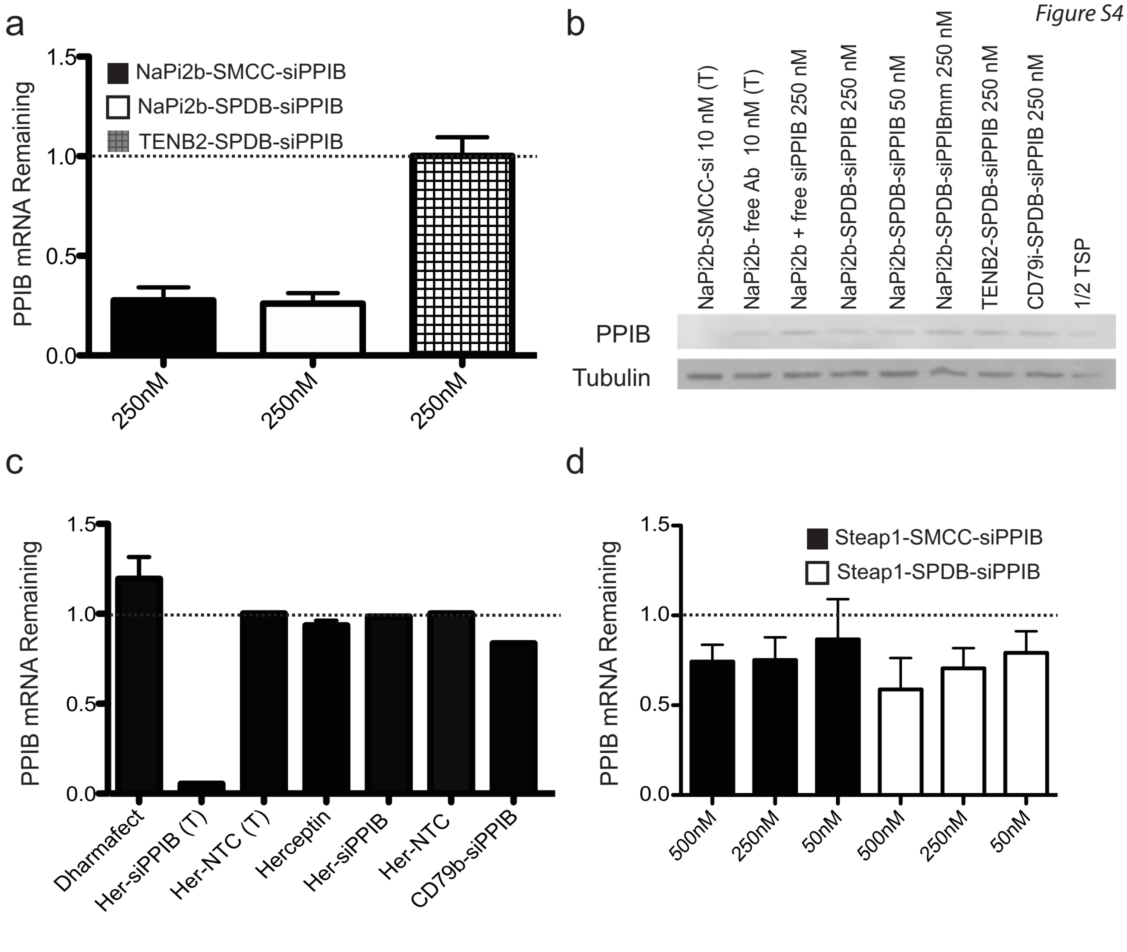
a
PPIB mRNA Remaining
1.5
1.0
0.5
0.0
NaPi2b-SMCC-siPPIB
NaPi2b-SPDB-siPPIB
TENB2-SPDB-siPPIB
250nM
250nM
250nM
b
NaPi2b-SMCC-si 10 nM (T)
NaPi2b- free Ab 10 nM (T)
NaPi2b + free siPPIB 250 nM
NaPi2b-SPDB-siPPIB 250 nM
NaPi2b-SPDB-siPPIB 50 nM
NaPi2b-SPDB-siPPIBmm 250 nM
TENB2-SPDB-siPPIB 250 nM
CD79i-SPDB-siPPIB 250 nM
1/2 TSP
PPIB
Tubulin
c
PPIB mRNA Remaining
1.5
1.0
0.5
0.0
Dharmafect
Her-siPPIB (T)
Her-NTC (T)
Herceptin
Her-siPPIB
Her-NTC
CD79b-siPPIB
d
PPIB mRNA Remaining
1.5
1.0
0.5
0.0
Steap1-SMCC-siPPIB
Steap1-SPDB-siPPIB
500nM
250nM
50nM
500nM
250nM
50nM

Figure S4. ARCs that bind NaPi2b and Steap1, but not Her2, mediate silencing. (a) Quantigene expression analysis of PPIB mRNA levels following a 72-hour administration of anti-NaPi2b ARCs to PC3-NaPi2b-High cells with non-reducible SMCC and reducible SPDB linkers (anti-TENB2 ARC was used as a negative control), N=4 for all samples, s.d. shown; samples were normalized to anti-NaPi2b-siNTC control ARCs (value set at 1.0). (b) Immunoblotting of PPIB protein in PC3-NaPi2b high cells following 72 hours of treatment with the indicated ARCs. T= lipid transfected controls; TSP = total soluble protein; CD79b and TENB2 = non-targeting control antibodies; free = use of unconjugated antibody or siRNA. (c) Gene expression analysis with the Quantigene assay shows PPIB levels following a 72-hour administration of Her2 ARCs to SKBR3 cells, which express extremely high levels of Her2 on the cell surface (Table 1) (N=4 for all samples, s.d. shown). Trastuzumab = free anti-Her2 antibody; CD79b = anti-CD79b non-targeting control antibody. (d) Quantigene expression analysis of PPIB mRNA levels following a 72-hour administration of Steap1 ARCs with the indicated linkers to 293 Steap1 cells (N=2 for all samples, s.d. shown).

Figure S5

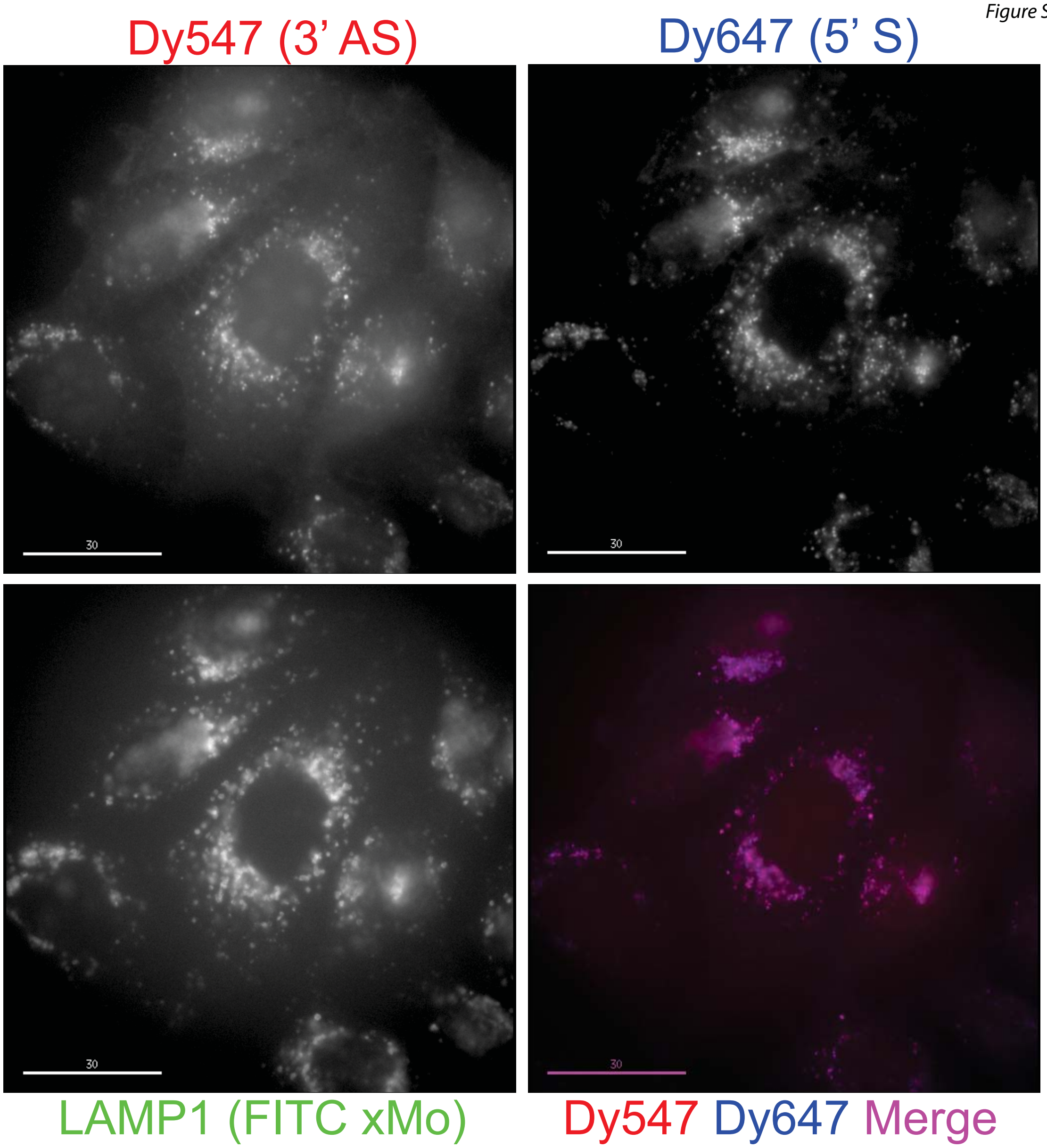
Dy547 (3' AS)
Dy647 (5' S)
30
30
30
30
LAMP1 (FITC xMo)
Dy547 Dy647 Merge

Figure S5. Pulse-chase experiment shows that ARCs deliver both siRNA strands to lysosomes. PC3-TENB2-High cells were treated for 30 min (pulse) with anti-TenB2-SMCC-PPIB ARCs, dual labeled with Dy647 (5' sense) and Dy547 (3' antisense). The medium was replaced, and cells were incubated for 18 hr without ARCs (chase). This pulse-chase design was used to reveal the destination compartment. Three panels show the individual localization patterns of each siRNA strand and LAMP1, as indicated; the patterns suggest co-localization of both siRNA strands with LAMP1. The fourth panel shows the merged image, with the sense strand in blue and the antisense strand in red; the magenta signal confirms complete co-localization.

Figure S6

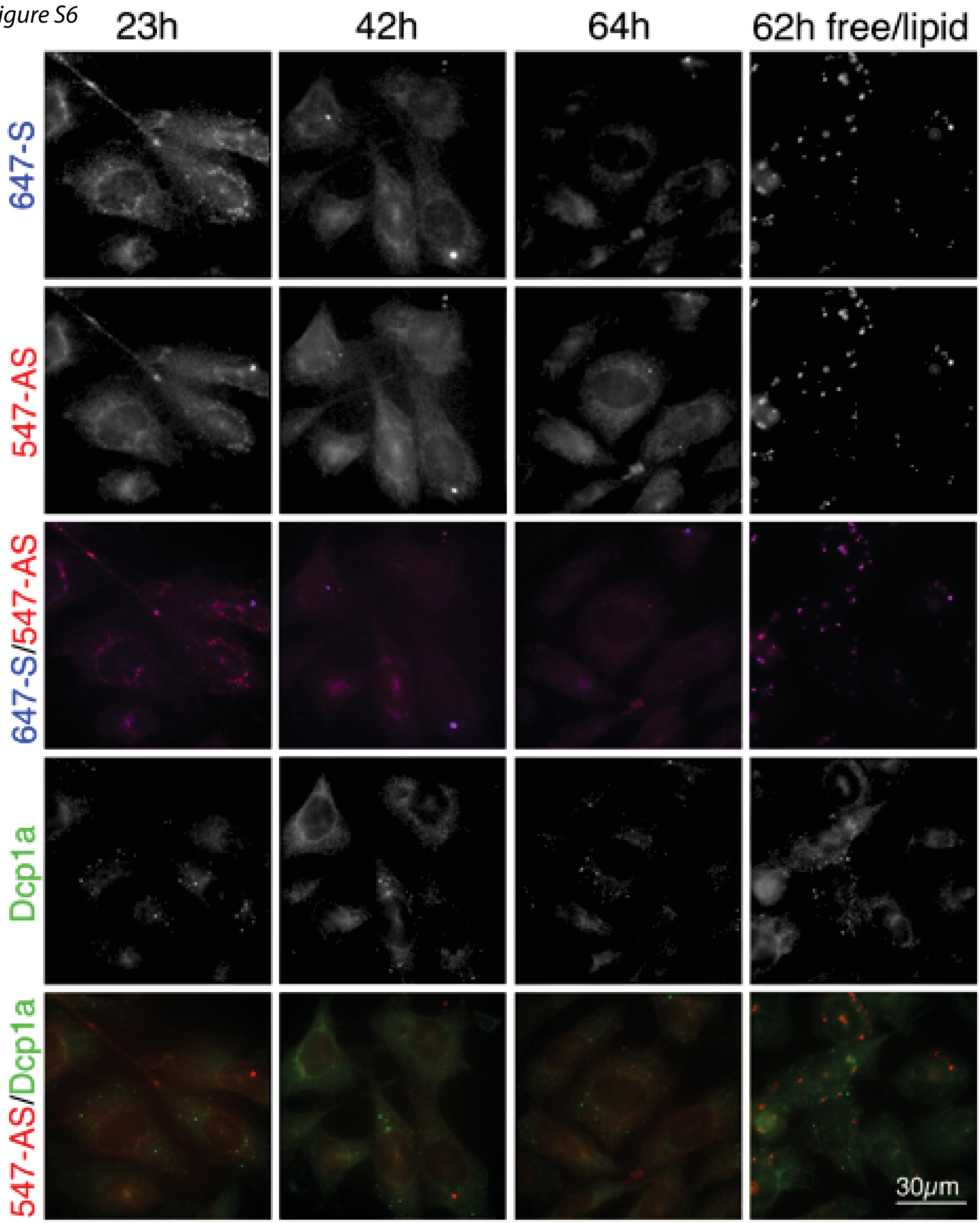
23h
42h
64h
62h free/lipid
647-S
547-AS
647-S/547-AS
Dcp1a
547-AS/Dcp1a
30μm

Figure S6. Pulse-chase experiment to assess ARC- and lipid-delivery of both siRNA strands to P-bodies. The experiment was performed as described in the legend to Supplementary Figure 5, except that the pulse was for 1 hr and the chases were done for the indicated times (three left columns); the localization patterns after 62 hr for free siRNA (not conjugated in an ARC) delivered by Liopfectamine are shown for comparison (right column). Dcp1a marks P-bodies (green).
